# Supplementary material for: Triggering of lymphocytes by CD28, 4-1BB, and PD-1 checkpoints to enhance the immune response capacities
Source: PLoS One. 2022 Dec 8;17(12):e0275777. doi: 10.1371/journal.pone.0275777 (PMC9731445; doi:10.1371/journal.pone.0275777)
Supplement: S1 Raw images — (PDF) [file pone.0275777.s001.pdf]

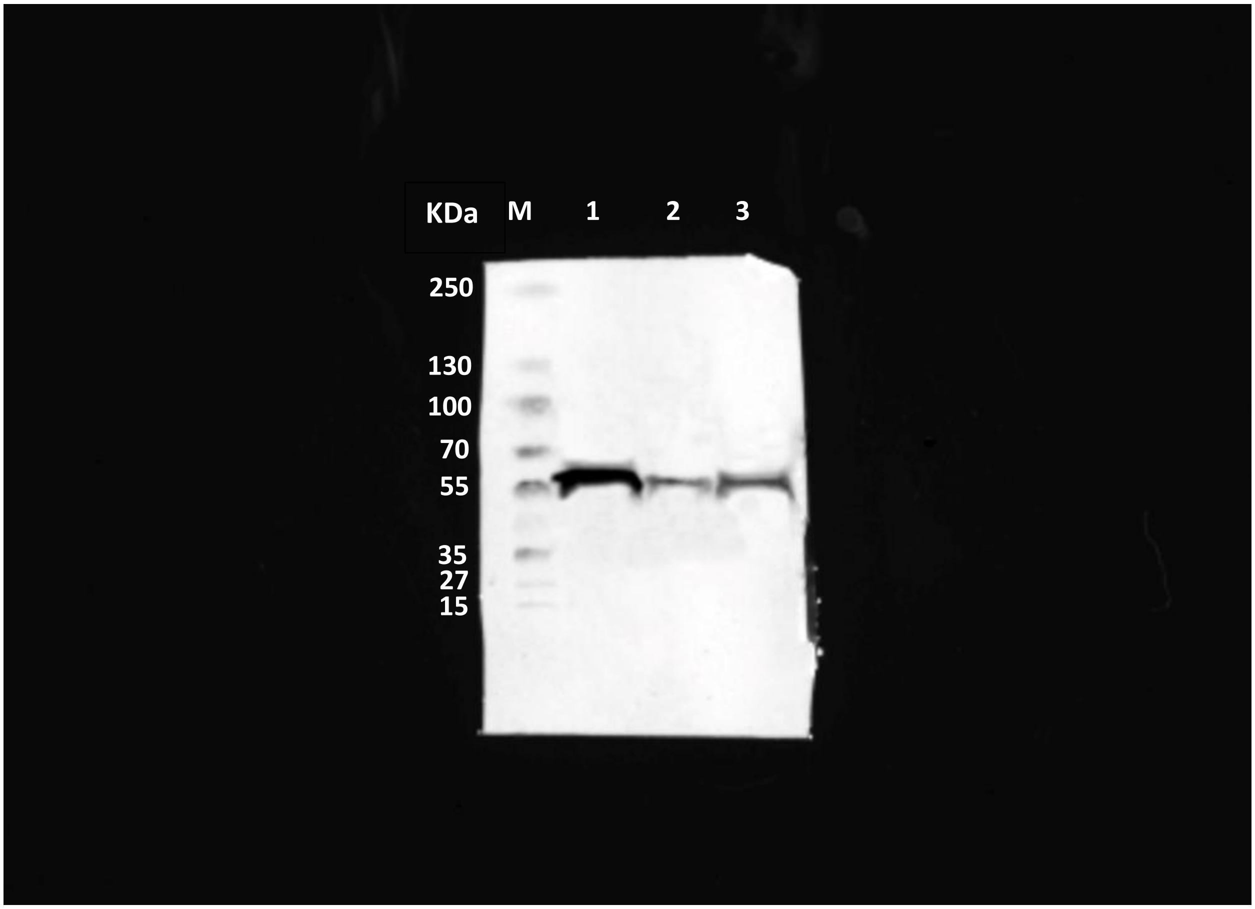

Figure Legend:

This is the original image of Fig. 1 (western blot).

Manipulation of blots: The image was cropped and also its quality was improved (increased resolution and adjusted brightness and contrast) using Photoshop software.

In original image you can observe the western blot analysis of purified Fc fusion proteins by probing with goat anti-human IgG (Fc specific)-peroxidase antibody under reducing conditions. Detection of peroxidase activity was performed by Enhanced Chemiluminescence (ECL) Reagents (Bio-Rad, USA). The immunoblot was imaged by ChemiDoc™ MP System (Bio-Rad, USA) and was analyzed with Image Lab™ Software. M: Protein ladder, Lane 1: anti- PD-1, Lane 2: CD80-Fc, Lane: 4-1BBL-Fc.
