# Supplementary material for: Triggering of lymphocytes by CD28, 4-1BB, and PD-1 checkpoints to enhance the immune response capacities
Source: PLoS One. 2022 Dec 8;17(12):e0275777. doi: 10.1371/journal.pone.0275777 (PMC9731445; doi:10.1371/journal.pone.0275777)
Supplement: S1 File — (PDF) [file pone.0275777.s002.pdf]

This is our study's minimal dataset file.

The means, standard deviations, standard errors, statistical method, P value, and replication of each condition in this research are reported. Indeed, these values were used to build graphs and reach the conclusion.

Fig. #	Mean	S.D	S.E	Statistical method used	P value #	samples
**Fig. 3B, M1**						
Responder-only	13.90	2.76	1.59	One way repeated measures ANOVA, Dunnett's test, Compere the mean of each column with mean of a untreated column	*p≤ 0.05 **p≤0.01	6
Untreated	24.26	4.24	2.45			6
PHA	38.33	1.5	0.88			6
Anti-PD-1	31.23	7.28	4.20			6
CD80-Fc	35.56	12.57	7.26			6
4-1BBL-Fc	34.3	13.27	7.66			6
Anti-PD-1/CD80-Fc	38.76	6.127	3.53			6
Anti-PD-1/4-1BBL-Fc	38.9	3.53	2.04			6
CD80-Fc/4-1BBL-Fc	40.5	5.51	3.18			6
Anti-PD-1/CD80-Fc/ 4-1BBL-Fc	41.13	4.04	2.33			6
**Fig. 3B,M2**						**Samples**
Responder-only	12.43	3.30	1.90			6
Untreated	20	8	4.6			6
PHA	30.75	5.05	2.9			6
Anti-PD-1	29.50	5.9	3.40			6
CD80-Fc	30.60	9	5.19			6
4-1BBL-Fc	27.95	6.45	3.72			6
Anti-PD-1/CD80-Fc	36.70	3.7	2.13			6
Anti-PD-1/4-1BBL-Fc	31.85	4.35	2.51			6
CD80-Fc/4-1BBL-Fc	38.25	2.95	1.70			6
Anti-PD-1/CD80-Fc/ 4-1BBL-Fc	40.40	1.7	0.981			6

Fig. #	Mean	S.D	S.E	Statistical method used	P value #	samples
**Fig. 4A**						
Untreated	26.22	1.95	1.12	One way repeated measures ANOVA, Dunnett's test, Compere the mean of each column with mean of a untreated column	*p≤ 0.05<br>**p≤0.01<br>***P≤0.0001<br>****P≤0.0001	6
Anti-PD-1	86.72	2.76	1.59			6
CD80-Fc	113.55	3.10	1.79			6
4-1BBL-Fc	114.79	5.21	3			6
Anti-PD-1/CD80-Fc	26.71	3.28	1.89			6
Anti-PD-1/4-1BBL-Fc	41.59	2.67	1.54			6
CD80-FC/4-1BBL-Fc	55.80	5.12	2.95			6
Anti-PD-1/CD80-Fc/ 4-1BBL-Fc	43.75	3.06	1.77			6
**Fig. 4B**						**Samples**
Untreated	17.17	2.24	1.29			6
Anti-PD-1	58.55	2.63	1.52			6
CD80-Fc	68.31	2.95	1.70			6
4-1BBL-Fc	64.76	1.75	1.01			6
Anti-PD-1/CD80-FC	16.82	2	1.15			6
Anti-PD-1/4-1BBL-Fc	22.83	2.95	1.70			6
CD80-FC/4-1BBL-Fc	32.41	2.77	1.60			6
Anti-PD-1/CD80-FC/ 4-1BBL-Fc	41.81	4.16	2.40			6
**Fig. 4C**						**Samples**
Untreated	1.42	0.51	0.29			6
Anti-PD-1	4.54	0.97	0.56			6
CD80-Fc	4.35	0.95	0.55			6
4-1BBL-Fc	3.64	0.713	0.41			6
Anti-PD-1/CD80-Fc	2.95	0.150	0.087			6
Anti-PD-1/4-1BBL-Fc	4.21	1.25	0.72			6
CD80-Fc/4-1BBL-Fc	4.63	1.15	0.66			6
Anti-PD-1/CD80-Fc/ 4-1BBL-Fc	3.44	0.60	0.35			6

Fig. #	Mean	S.D	S.E	Statistical method used	P value #	samples
**Fig. 4D**						
Untreated	2.31	0.84	0.48	One way repeated measures ANOVA, Dunnett's test, Compere the mean of each column with mean of a untreated column	*p≤ 0.05	6
Anti-PD-1	3.66	1.15	0.66			6
CD80-Fc	2.89	1.40	0.80			6
4-1BBL-Fc	3.06	0.97	0.56			6
Anti-PD-1/CD80-Fc	2.79	1.50	0.86			6
Anti-PD-1/4-1BBL-Fc	3.556	1.45	0.83			6
CD80-Fc/4-1BBL-Fc	3.98	1.56	0.90			6
Anti-PD-1/CD80-Fc/ 4-1BBL-Fc	3.39	0.85	0.49			6
**Fig. 4E**						**Samples**
Untreated	2.72	1.16	0.48			6
Anti-PD-1	3.65	0.93	0.66			6
CD80-Fc	3.95	0.55	0.80			6
4-1BBL-Fc	4.03	0.85	0.56			6
Anti-PD-1/CD80-Fc	2.56	0.81	0.86			6
Anti-PD-1/4-1BBL-Fc	3.39	0.90	0.83			6
CD80-Fc/4-1BBL-Fc	3.42	1.09	0.90			6
Anti-PD-1/CD80-Fc/ 4-1BBL-Fc	2.04	0.566	0.49			6
**Fig. 4F**						**Samples**
Untreated	10.36	1.93	1.11			6
Anti-PD-1	10.51	2.50	1.44			6
CD80-Fc	10.43	1.33	0.76			6
4-1BBL-Fc	10.67	1.23	0.71			6
Anti-PD-1/CD80-Fc	10.28	1.43	0.83			6
Anti-PD-1/4-1BBL-Fc	10.38	1.09	0.63			6
CD80-Fc/4-1BBL-Fc	10.37	1.60	0.92			6
Anti-PD-1/CD80-Fc/ 4-1BBL-Fc	10.13	1.84	1.06			6

Fig. #	Mean	S.D	S.E	Statistical method used	P value #	Samples
**Fig. 5A (1:1)**						
Untreated	7.71	3.88	1.58	Two-way ANOVA, Dunnett's test, Compere each cell mean with the control mean on that row	*p≤ 0.05 **p≤0.01 ***P≤0.0001	6
PHA	12.54	7.02	2.86			6
Anti-PD-1	10.25	5.5	2.24			6
CD80-Fc	10.98	6.73	2.75			6
4-1BBL-Fc	13.87	5.98	2.44			6
Anti-PD-1/CD80-Fc	13.11	7.11	2.90			6
Anti-PD-1/4-1BBL-Fc	15.27	6.46	2.64			6
CD80-FC/4-1BBL-Fc	18.88	10.07	4.11			6
Anti-PD-1/CD80-Fc/ 4-1BBL-Fc	16.97	5.42	2.21			6
**Fig. 5A (1:10)**						**Samples**
Untreated	7.808	2.259	0.922			6
PHA	15.17	41.16	1.69			6
Anti-PD-1	16.96	4.36	1.78			6
CD80-Fc	14.45	3.99	1.63			6
4-1BBL-Fc	19.23	5.007	2.04			6
Anti-PD-1/CD80-Fc	18.6	5.43	2.18			6
Anti-PD-1/4-1BBL-Fc	21.03	5.9	2.4			6
CD80-FC/4-1BBL-Fc	18.51	6.23	2.54			6
Anti-PD-1/CD80-Fc/ 4-1BBL-Fc	17.36	3.62	1.47			6

Fig. #	Mean	S.D	S.E	Statistical method used	P value #	samples
**Fig. 5B**						
Untreated	7.808	2.259	0.922	One way repeated measures ANOVA, Dunnett's test, Compere the mean of each column with mean of a untreated column	*p≤ 0.05 **p≤0.01 ***P≤0.0001	6
PHA	15.17	41.16	1.69			6
Anti-PD-1	16.96	4.36	1.78			6
CD80-Fc	14.45	3.99	1.63			6
4-1BBL-Fc	19.23	5.007	2.04			6
Anti-PD-1/CD80-FC	18.6	5.43	2.18			6
Anti-PD-1/4-1BBL-Fc	21.03	5.9	2.4			6
CD80-FC/4-1BBL-Fc	18.51	6.23	2.54			6
Anti-PD-1/CD80-Fc/ 4-1BBL-Fc	17.36	3.62	1.47			6
