# Supplementary material for: Triggering of lymphocytes by CD28, 4-1BB, and PD-1 checkpoints to enhance the immune response capacities
Source: PLoS One. 2022 Dec 8;17(12):e0275777. doi: 10.1371/journal.pone.0275777 (PMC9731445; doi:10.1371/journal.pone.0275777)
Supplement: Attachment — Submitted filename: Response to Reviewers.docx [file pone.0275777.s004.docx]

We appreciate the reviewers' and editor's insightful comments, which helped strengthen our manuscript. All comments were accepted and the appropriate changes to the manuscript were made using “track changes.” We believe that these revisions have greatly improved the manuscript, and we hope that you will now consider it suitable for publication in **PLOS ONE**. Our point-by-point detailed responses to comments are listed on the following pages.

**Response to Editors:**

**Editors:** *Please note that funding information should not appear in any section or other areas of your manuscript. We will only publish funding information present in the Funding Statement section of the online submission form. Please remove any funding-related text from the manuscript.*

**Authors:** In response to the respected editor's comment, the “Funding information” was removed from the manuscript.

**Editors:** *Your ethics statement should only appear in the Methods section of your manuscript. If your ethics statement is written in any section besides the Methods, please move it to the Methods section and delete it from any other section. Please ensure that your ethics statement is included in your manuscript, as the ethics statement entered into the online submission form will not be published alongside your manuscript.*

**Authors:** The separate part titled "Ethics statement" at the end of the Conclusion section was removed and entered in the Methods section. Lines 157-160 (in the “Functional analysis” part) contained all the necessary details and explanations related to the ethics statement.

“Fresh whole peripheral blood was prepared from healthy human donors, and PBMCs were isolated using Ficoll density gradient separation. Donors were male and female, aged 25 to 45 years. Notably, each PBMC donor signed informed consent prior to blood collection. All procedures were verbally and in writing explained to them prior to signing the form, and all questions were fully answered. **Also, the study protocols were reviewed and approved by the research ethics committee of Shiraz University of Medical Sciences (Approval ID: IR.SUMS.REC.1398.938). None of the PBMCs donors were from a vulnerable population, and all donors or next of kin provided written informed consent that was freely given.**”

**Response to Reviewer:**

**Reviewer 1:** *Please authors describe how the 30 Gy was determine for γ-irradiation of PBMCs?*

**Authors:** Previous studies in MLR assays have shown that stimulator cells are exposed to high doses of gamma irradiation (typically 30 Gy= 3000 rad) to prevent proliferation while maintaining their ability to stimulate the proliferation and cytokine profiles of allogeneic T cells. Tourkova et al., 2001 reported that irradiated (30 Gy) and non-irradiated DCs had similar stimulatory effects. As a result, these findings suggest that irradiating stimulator cells with a 30 Gy dose had no effect on their ability to induce T cell proliferation in MLR. Therefore, in this study, 30 Gy radiation was chosen. To confirm the irradiation process efficacy, the stimulator PBMCs were stained with CFSE and cultured in the presence of PHA (1:1000 (v/v)). After 5 days, the cells were harvested and stained with 7-AAD, and a cell proliferation test was performed to confirm the accuracy of the amount of radiation used; the cells were found to be alive but unable to divide.

The relevant references have been included in the manuscript.

- Tourkova IL, Yurkovetsky ZR, Shurin MR, Shurin GV. Mechanisms of dendritic cell-induced T cell proliferation in the primary MLR assay. Immunology letters. 2001;78(2):75-82.
- Pourfathollah A-A, Shaiegan M, Namiri M, Babae G-R. Effect of gamma irradiation on lymphocyte proliferation and IL-8 production by lymphocytes isolated from platelet concentrates. Archives of medical research. 2008;39(6):590-3.

**Reviewer 1:** *Please clarify the statement: (non-proliferative but potentially antigen-presenting cells)*

**Authors**: The following explanation was added to the manuscript, along with relevant references:

“Irradiation prevents the proliferation of stimulator cells by inhibiting DNA replication but has no effect on transcription, preserving their ability to induce allogeneic T cells proliferation”.

**Reviewer 1:** *Image (1and 4) quality need to improve*

**Authors**: In response to the reviewer's comment, the resolution of Figure 1 was increased. As a result, a new "**S1 raw images**" file was provided as a Supporting Information file. This Figure was imaged by ChemiDoc™ MP System (Bio-Rad, USA) and was analyzed with Image Lab™ Software. Figure 4's quality was also improved.

**Reviewer 1:** *Histogram plots in Figure 3A quality is poor, Please replace the images with higher resolution images.*

**Authors:** A higher resolution image was used to replace Figure 3.

**Reviewer 1:** *The caption need clarification” (A) Histogram plots show M1 (divided) and M2 (highly 270 divided) populations of untreated (Light gray) and PHA conditions (dark gray)”*

**Authors**: The caption was edited; “Histogram plots depict examples of two populations used in this study for MLR analysis: M1 (divided) and M2 (highly divided) populations of untreated (Light gray) and PHA conditions (dark gray). M2 is a subset of the M1 population that contains cells with a high division rate.”

**Reviewer 1:** *The results of* *T cell Proliferation (The M1 and M2 population) should be explained in detail in discussion.*

**Authors**: In response to the respected reviewer's comment, additional explanations about T cell Proliferation (The M1 and M2 population) were added to Result and Discussion sections:

*The Result Part:* “Indeed, the M2 is a subset of the M1 population that contains cells with a high division rate. The results of the statistical significance (p-value of <0.05) were the same for both populations (Fig 3B).”

*The Discussion Part:*” In order to analyze the MLR results, M1 (divided) and M2 (highly divided) populations were considered. The statistically significant results (p-value of 0.05) of the two populations were the same, suggesting that the M2 population is primarily responsible for the proliferation results in this study.”
